# IX international conference on Salt Lake research: Research opportunities and management challenges

**DOI:** 10.1186/1746-1448-1-12

**Published:** 2005-12-28

**Authors:** Robert Jellison

**Affiliations:** 1 Marine Science Institute, University of California, Santa Barbara, CA 93106-6150 USA

## Abstract

The 9th International Conference on Salt Lake Research was held 26–30 September 2005 in Western Australia at the Curtin University of Technology, Perth, Australia. One hundred scientists from 10 countries presented research on a diverse array of topics highlighting research findings and opportunities, and management challenges associated with inland saline waters. Major emergent themes of the conference included modeling of ecosystem processes, microbial communities, and features of Western Australian inland saline environments, including current threats, conservation and management.

## Background

**Figure 1:**
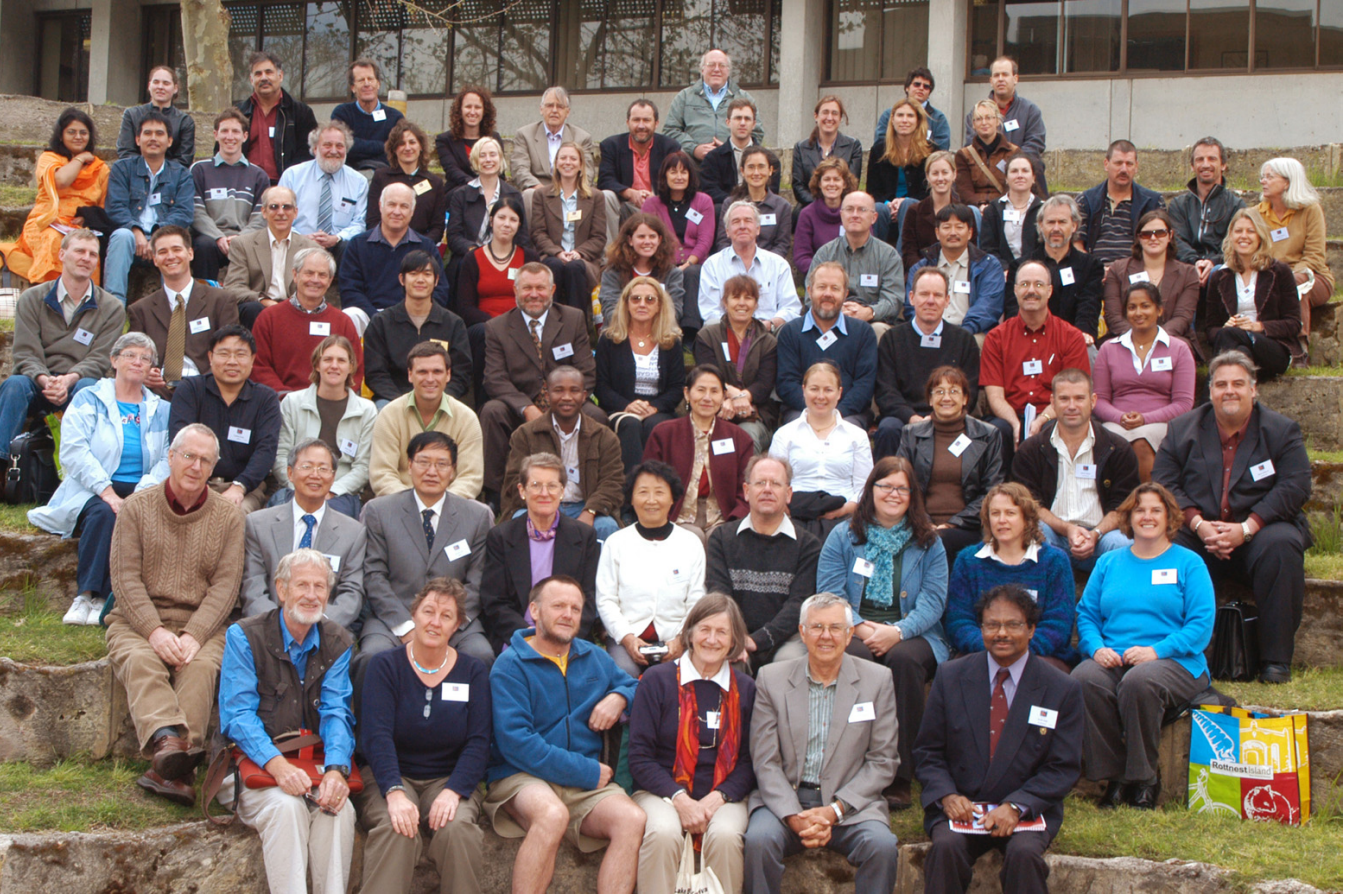
Participants of the IX Int. Conf. Salt Lake Research.

Since 1979 the International Society for Salt Lake Research has organized triennial conferences in various salt lake regions of the world including the USA, Canada, China, Russia, Kenya, Bolivia, Spain, and South Australia [1]. The 9th International Conference on Salt Lake Research was held 26–30 September 2005 in Western Australia at the Curtin University of Technology, Perth, Australia. One hundred scientists from 10 countries presented research on a diverse array of topics highlighting research findings, opportunities, and management challenges associated with inland saline waters. (Figure 1)

Salt lakes, typically defined as standing waters with salinity greater than 3 g l^-1^, and other inland saline habitats encompass a diverse array of ecosystems and the 3-day scientific program was equally wide-ranging. While this brief commentary cannot do justice to all the topics presented as part of the scientific program (see abstracts online [2]), I consider several emergent themes of the conference before finishing with some general remarks.

## Results and discussion

### Modeling ecosystem processes in permanent salt lakes

Permanent salt lakes lie along a continuum of salinity and all but those at the highest salinities share many common features and processes with their freshwater counterparts. They also share common threats and anthropogenic stressors with eutrophication, toxic contamination, exotic species invasions, and over-fishing among the most important. Of the widespread stressors on freshwater lakes, changing hydrological and salinity regimes caused by water diversions and climate change are major concerns, threatening permanent salt lakes throughout the world [3]. By contrast, acid precipitation is of relatively minor concern to salt lakes. As with freshwater lakes, complex ecological models of salt lakes are an essential tool in predicting ecological responses to stressors and various management actions. Thus, the opening plenary session by Jorg Imberger entitled "A practitioner's view of modern developments in limnology" was fitting and well-received. The presentation described the real-time application of coupled hydrodynamic-ecological models in coordination with sophisticated *in situ *instrumentation for designing process-oriented limnological experiments.

Developing complex ecological models is a large task and Imberger has been central to the development, implementation, and maintenance of a suite of hydrodynamic and ecological models at the Centre for Water Research, UWA (CWR) [4]. CWR's long-term commitment to the development, validation, and maintenance of these models has led to their use by researchers in 59 countries. They are valuable tools, yet have until recently been underutilized by salt lake researchers, and this was a good forum for greater exposure. In cases where salt lakes are managed for specific flora and fauna or more general ecosystem properties, models will likely prove valuable tools. In the past, salt lakes were insufficiently studied and understood to develop complex ecological models. However, given the increased study of many large salt lakes (e.g. Shira Lake, Mono Lake, Salton Sea, Great Salt Lake, and Walker Lake) the time is ripe for modeling analyses aimed at furthering our understanding of lake-level processes, directing new research, and designing management regimes. Significantly, presenters used complex lake models to examine the role of zooplankton in the ecological succession of plankton and benthic algae across a salinity gradient in Shark Bay solar salt ponds and to model sulfur bacteria in meromictic Lake Shira.

It has often been hypothesized that the lower biodiversity in moderately saline and hypersaline lakes should make them more amenable to modeling than their more biotically complex freshwater counterparts. This has not yet been tested and even simple systems may display complex behaviors as shown by presentations on *Artemia *juvenile bottlenecks and alternative stable states of shallow saline lakes. However, modeling of simpler saline lake ecosystems will likely provide insights into the challenges of modeling more complex freshwater lakes.

### Microbial research in inland saline environments

The advent of new molecular tools and recognition of the predominant role of microbes in biogeochemical fluxes and cycling, which control or effect nearly all ecosystem processes, have led to an increasing interest in the microbes of saline environments. The unique species and high metabolic diversity of microbes associated with the chemically diverse array of saline environments presents excellent opportunities for basic research. In the US, the National Science Foundation's Microbial Observatories research initiative includes many saline environments (e.g. Soap Lake, Mono Lake, Cabo Rojo salterns, McMurdo Dry Lake Valley, salt marshes, microbial mats, hypersaline lagoons, and Salt Plains). New species with unique metabolic abilities are routinely described and the conference included a good sampling of these activities from around the world.

A plenary talk by Juergen Wiegel (Univ. Georgia, USA) entitled "Life at extreme limits: halophilic alkalithermophiles, a novel group of extremophiles" began the second day of presentations. These "triple" extremophiles form a unique group of organisms growing in physiochemical conditions beyond those currently described for microbial growth. Microbial research from the Great Salt Lake, Mongolian soda lakes, gypsum crusts in salterns, meromictic Shira Lake, and microbial mats of Rottnest Island (Western Australia) salt lakes was presented.

### Western Australian saline environments – features, threats, and management

Jacob John (Curtin Univ., Perth, AUS), the conference host and chief organizer, presented an overview of saline environments in Western Australia with associated threats and conservation issues in the third plenary session. Western Australia is rich in saline environments including coastal and island salt lakes, hypersaline lagoons containing stromatolites and thrombolites, large inland salt lakes, and thousands of small seasonally filled and episodic ephemeral salt lakes. John and colleagues presented papers on the diatom flora of microbial mats, acid lakes, the Canning River, and a regional study of rivers and lakes which developed a novel diatom transfer function for detecting secondary salinisation.

Despite their widespread occurrence, inland saline environments have historically received much less attention than their freshwater counterparts and there is much work yet to be done to adequately describe those of Western Australia. This was aptly illustrated during the post-conference field trip, when we visited lakes containing four undescribed species of *Parartemia*! One was discovered only a couple of weeks before during scouting for the field trip. The other three have been known for a few years, but their specific habitat requirements are just being learnt. It is a race against time to describe their diversity and environmental adaptations before their habitats are lost to secondary salinisation. Descriptive research on many aspects of Western Australia's inland saline environments provided conference participants with an appreciation of the regional natural diversity. Groundwater estuaries, regions of inland subterranean salinity gradients, containing diverse and ancient fauna represented one of the more unusual ones. Other regions of Australia were also discussed, including descriptions of lakes and their fauna on the Eyre Peninsula (South Australia) and the middle Paroo (New South Wales) by Brian Timms.

Secondary salinisation is one of the chief threats facing inland rivers and lakes in large agricultural regions of Australia and, although less well-documented elsewhere, it also occurs in other parts of the world [5,6]. This process involves mobilizing and transporting salts dissolved in underground water to the surface by rising groundwater associated with decreased transpiration following removal of deep-rooted vegetation or excessive irrigation and poor drainage. Many aspects of this widespread problem were discussed including floral and faunal inventories across impacted communities, detection and monitoring, hydrology, risk management, conservation and restoration strategies.

Further inland, salt loads are increased by large-scale mining operations that discharge hypersaline groundwater into surface lakes and drainages. Several researchers addressed monitoring, which is made more difficult by the episodic nature of these systems, and the actual and potential impacts of these discharges.

### Conservation of salt lakes, the Ramsar Treaty, and avian habitat

As economic forces are driving the destruction of many ecosystems on which human well-being depends, a broad and rapidly growing field of eco-economics has arisen with the goal of building a worldwide sustainable economy [7]. Proponents hope to harness market forces in restructuring the world economy by assessing ecosystem services and developing policies which internalize the costs of environmental destruction. However, given the high economic value of fresh water and the current and pending crises in availability, most permanent salt lakes are likely to continue to undergo significant desiccation and salinisation. The fate of most permanent salt lakes will likely be decided in the next few decades.

While many permanent salt lakes have significant economic value (e.g. mining, fisheries, recreation), in many, if not most cases, the economic value of water diverted for agriculture or industrial and human consumption may be much greater. Cultural, wildlife, especially avian, biodiversity, aesthetic, and other non-economic values are often defining features of salt lakes. Any economic assessment of these values is difficult and dependent on cultural and human conditions. Where salt lake conservation has occurred it is often because of increased awareness and valuation brought about by nongovernmental organizations and advocacy groups.

Since 1971, the International Treaty on Wetlands (Ramsar Treaty) has been instrumental in raising the public awareness of the ecological value of all types of wetlands including inland saline lakes [8]. Of 1,477 sites in 147 countries, 372 (25%) include inland saline environments. Australia leads the world with 64 designated Ramsar sites of which 26 (41%) include inland saline environments. Although the conference included many presentations on ecological impacts and several on research conducted at Ramsar sites, unfortunately no direct mention of the efficacy or impact of Ramsar status was noted. An accounting of the experiences with Ramsar sites as conservation tools would be informative for other countries at future meetings.

The most widely recognized ecological value of salt lakes is as habitat for migratory and nesting populations of birds. While the critical dependence of flamingo populations throughout the world on salt lakes is widely publicized, many other waterbirds are equally dependent on salt lakes, including many species of phalaropes, grebes, gulls, pelicans, swans, and plovers. All 26 of the Australian Ramsar sites are listed as important to birds. Hindle and colleagues combined bird monitoring and habitat mapping to develop a model of Black Swan feeding habitat for monitoring and management. Others presented papers on avian use at saline wetlands in eastern Australia, and San Francisco estuaries and Salton Sea of California.

### Aquaculture and diverse other topics

Aquaculture is playing an increasing role in feeding the world as demand increases and natural fishery stocks are depleted. In China, fish farm output is about 30% of the oceanic fish catch [9]. A suite of presentations by Curtin University researchers looked at the feasibility of aquaculture in inland saline waters of Western Australia.

Finally, other presentations addressing a wide range of topics illustrative of the diverse topics presented at the meeting, including, the roles of calcium and iron in Northern Great Plains lakes (USA), sulfide irruption and gypsum blooms in the Salton Sea, and hydrochemical characteristics of salt lakes in the Qinhai-Tibet region.

### ISSLR conferences

ISSLR was founded to enhance communication and interaction among scientists interested in inland saline waters, to encourage these interests, and to educate the public in the scientific management and conservation of salt lakes. Organizing a triennial salt lake conference and editing volumes of selected papers are primary activities of the society. Conference sites are purposefully selected to highlight various salt lake regions of the world, engage regional scientists and managers, and enable field trips to the diverse array of aquatic habitats represented by inland saline lakes.

The Perth conference was well-organized and the venue well-suited to a small scientific meeting. Following brief welcoming remarks by Prof. Lance Twomey, Vice-Chancellor and President of Curtin University and Hon. Dr. Judy Edwards, Minister for the Environment and Science (Western Australia), the Wadumbah Aboriginal Dance Group treated participants to traditional music and dances. Mid and post-conference field trips enabled participants to visit local and regional saline habitats. ISSLR's president and fairy shrimp enthusiast, Brian Timms (Univ. Newcastle, New South Wales) led an unforgettable 7-day post-conference field trip inland through a vast array of inland salt lakes and back along the southwest coast.

The late W. D. Williams was a driving force in Australian limnology and passionately interested in salt lake research and conservation. In his honor, the first Williams' awards for superior student presentations were awarded at the Perth conference. As a member of the award committee, I was particularly pleased with the quality and number of student presentations. Recipients included Michelle Hindle (Univ. Wollongong, NSW), Lien Sim (Murdoch Univ., WA) and Courtney Salm (Univ. Wisconsin, La Crosse, WI, USA) presenting research on Black Swan feeding habitat, submerged plants in salinised wetlands, and primary production in prairie saline lakes of the USA, respectively (see conference abstracts for further information).

## Conclusion

Inland saline ecosystems present unique research opportunities and pressing conservation and management issues. The research spans much of modern limnology and progress requires integration and interaction with the larger limnological community represented by ASLO, SIL, national limnological societies, and the various subdisciplines. Saline Systems, whose primary aim is to enhance communication between and among scientists concerned with saline environments, is strongly supported by many ISSLR members as evidenced by the editorial board (half of whom are active ISSLR members) and manuscript submissions. The open access format should facilitate sharing knowledge among scientists, managers, and policy-makers throughout the world to aid conservation and sustainable management.

ISSLR has maintained a policy of holding each of their triennial conferences within a different salt lake region, sometimes at remote locations away from major transportation hubs. As a result, these conferences have engaged scientists from diverse backgrounds and participants gain new perspectives on regional management challenges and research opportunities. They are usually held in intimate and often spectacular settings, and have resulted in memorable experiences of both high scientific merit and personal value. Extended post-conference field trips provide participants with first-person experiences and knowledge of saline environments that cannot be gained by other means. The Perth conference fulfilled all these expectations and having attended this and previous conferences, it is clear to me they occupy a special niche that many scientists find rewarding. I look forward to the next and many more.
